# A promoter library for tuning gene expression in *Cupriavidus necator* under autotrophic conditions

**DOI:** 10.3389/fbioe.2025.1595440

**Published:** 2025-07-04

**Authors:** Wataru Kitagawa, Kensuke Igarashi, Ryo Nagasawa, Shigeyuki Kakizawa, Mizuki Horino, Kosuke Fujishima, Toshiaki Fukui, Souichiro Kato

**Affiliations:** ^1^ Biomanufacturing Process Research Center, National Institute of Advanced Industrial Science and Technology (AIST), Sapporo, Japan; ^2^ Division of Applied Bioscience, Graduate School of Agriculture, Hokkaido University, Sapporo, Japan; ^3^ Molecular Biosystems Research Institute, National Institute of Advanced Industrial Science and Technology (AIST), Tsukuba, Ibaraki, Japan; ^4^ School of Life Science and Technology, Institute of Science Tokyo, Tokyo, Japan; ^5^ Earth-Life Science Institute, Institute of Science Tokyo, Tokyo, Japan; ^6^ Graduate School of Media and Governance, Keio University, Fujisawa, Japan; ^7^ School of Life Science and Technology, Institute of Science Tokyo, Yokohama, Kanagawa, Japan; ^8^ Research Center for Solar Energy Chemistry, Graduate School of Engineering Science, Osaka University, Osaka, Japan

**Keywords:** *Cupriavidus necator*, CO_2_ fixation, biomanufacturing, transcriptome, promoter library, heterologous expression

## Abstract

*Cupriavidus necator* holds promise for biomanufacturing using CO_2_ as the primary feedstock, leveraging its capabilities to produce valuable chemicals and grow autotrophically using H_2_ as an energy source. Although various genetic tools, including promoters, have been developed to fine-tune gene expression in *C. necator*, no such tools have been developed for the use in autotrophic conditions. This study aimed to establish a promoter library that functions in *C. necator* grown under autotrophic conditions. *C. necator* was cultured under both heterotrophic and autotrophic conditions, and comparative transcriptome analysis was performed to identify genes/operons specifically upregulated under autotrophic conditions and those constitutively expressed. The upstream sequences of the candidate genes/operons were examined to identify their promoter regions. We established a promoter evaluation system based on colorimetric measurement of β-galactosidase activity in *C. necator*. Utilizing this system, we successfully identified seven promoters that specifically upregulate the downstream gene encoding β-galactosidase under autotrophic conditions and three promoters that constitutively express the gene under both autotrophic and heterotrophic conditions. We designed expression gene cassettes in which exogenous genes are placed downstream of the autotrophic-specific promoters and constructed a *C. necator* strain with the gene cassettes inserted into the genome. Quantitative RT-PCR analysis confirmed the expression of the exogenous genes under autotrophic conditions. This study represents the first development of a promoter library that functions in *C. necator* under autotrophic conditions without the need for specific external inducers. This advancement lays the groundwork for more efficient CO_2_-based biomanufacturing platforms, contributing to the development of sustainable bioprocesses.

## 1 Introduction

Biomanufacturing, a biotechnology that utilizes biological systems for the synthesis of commercially relevant compounds, has garnered significant attention due to its energy efficiency, reduced dependence on fossil resources, and its pivotal role in fostering a sustainable economy (Clomburg et al., 2017; Zhang et al., 2017). Conventional biomanufacturing has mainly relied on edible organics, such as sugars, proteins and oils, derived from cultivated crops. However, concerns of competition with food, land use issues, depletion of water resources, etc., necessitate the exploration of more sustainable feedstock alternatives (Alalwan et al., 2019; Scown, 2022). In addition to utilizing non-edible biomass (Singh et al., 2022) and algal biomass (Sørensen et al., 2022), biomanufacturing processes that use CO_2_ as a primary feedstock are garnering substantial interest (Salehizadeh et al., 2020; Bachleitner et al., 2023). Autotrophic microorganisms, capable of utilizing electricity, H_2_, CO, and other energy sources for CO_2_ fixation, are employed as biocatalysts for CO_2_-based biomanufacturing (Igarashi and Kato, 2017; Kurt et al., 2023).

*Cupriavidus necator* (formerly known as *Ralstonia eutropha*) is a promising bacterium for CO_2_-based biomanufacturing due to its ability to produce useful chemicals and CO_2_ fixation capacity (Panich et al., 2021; Tang et al., 2023; Weldon and Euler, 2025). *C*. *necator* has a natural biosynthetic pathway for producing the biodegradable polymer poly(3-hydroxybutyrate). The genetic modification and metabolic engineering of *C. necator* have been extensively investigated to enhance the efficient production of practical biopolymers (Koller and Mukherjee, 2022; Tang et al., 2022; Morlino et al., 2023) and to facilitate the biosynthesis of other valuable compounds, such as biofuels (Chakravarty and Brigham, 2018). Although biomanufacturing using *C. necator* has relied on edible sugars and oils derived from cultivated crops, there is a significant demand for more sustainable feedstocks (Zhang et al., 2022). The ability of *C. necator* to grow autotrophically using H_2_ as an energy source is expected to enable the CO_2_-based biomanufacturing. In fact, it has been reported that *C. necator* has ability to produce biopolymers from CO_2_ (Ishizaki and Tanaka, 1991), and the productivity can be enhanced through genetic engineering, such as overexpressing the CO_2_-fixing pathway (Kim et al., 2022) and the carbonic anhydrase (Thorbecke et al., 2021), as well as reactor engineering (Tanaka et al., 2023; Di Stadio et al., 2024).

The practical application of CO_2_-based biomanufacturing requires engineered *C. necator* strains that can efficiently produce the target compounds under autotrophic conditions. Although synthetic biology toolkits such as genetic engineering vectors, transformation methods, genome engineering techniques, and information of central and peripheral metabolic pathways are available, promoters suitable for autotrophic growth conditions are limited. While promoters that function in *C. necator* have been extensively explored and developed, they were designed for use under heterotrophic and/or PHA-producing conditions (Fukui et al., 2011; Alagesan et al., 2018; Johnson et al., 2018; Pan et al., 2021; Mishra et al., 2024; Santolin et al., 2024; Wang et al., 2024). Several research groups have reported the expression of exogenous genes in *C. necator* under autotrophic conditions (Thorbecke et al., 2021; Kim et al., 2022; Arhar et al., 2024; Panich et al., 2024). The promoters used in these studies include constitutive and inducible promoters functioning across diverse microbial species (*lac* promoter [P_*lac*_] and *araBAD* promoter [P_BAD_], respectively), as well as endogenous promoters expected to function robustly under autotrophic conditions (*cbb* promoter, regulating gene clusters of the Calvin-Benson-Bassham [CBB] cycle enzymes). There has been no research on comprehensive exploration of promoters capable of fine-tuning gene expression in *C. necator* under autotrophic conditions, which is essential for the practical implementation of CO_2_-based biomanufacturing processes.

In this study, we aimed to develop a promoter library for *C. necator* that functions under autotrophic conditions. The gene expression of *C. necator* was compared under heterotrophic and autotrophic growth conditions to identify candidate promoters. The activities of the candidate promoters were assessed by β-galactosidase expression analysis and quantitative real-time RT-PCR (qRT-PCR) analysis. We successfully identified seven promoters that specifically upregulate downstream genes under autotrophic conditions and three promoters that constitutively express downstream genes regardless of culture conditions.

## 2 Materials and methods

### 2.1 Bacterial strains and culture conditions

The bacterial strains used in this study are listed in Table 1. *Cupriavidus necator* and *E. coli* strains were routinely cultured in a Luria-Bertani (LB) medium (Kato et al., 2017) at 30°C and 37°C, respectively, with agitation at 120 rpm. When necessary, kanamycin (50 mg/L), chloramphenicol (34 mg/L), or ampicillin (50 mg/L) was added to the medium.

**TABLE 1:** Bacterial strains used in this study.

Bacterial strain	Description	Reference
*Cupriavidus necator*		
H16	Wild type, PHA+, non-glucose assimilation	DSM 428
IP-015	H16 derivative, ∆*phaC ∆phaB1 ∆phaB3* ∆*nagR nagE*(G793C) *∆paaH1 ∆had ∆phaR ∆phaP1::adh-adc*, PHA-, glucose assimilation, isopropanol production	Subagyo et al. (2021)
IP015DL	IP-015 derivative, containing *lox71*, *cmr*, *lox m2/66* on the genome at the locus tag of H16_A0404	This study
DL_1A23	IP-015DL derivative, containing Em-CoA pathway genes	This study
*Escherichia coli*		
XL1-Blue	*hsdR*17, *supE*44, *recA*1, *endA*1, *gyrA*46, *thi, relA*1, *lac*/F' [*proAB* ^+^, *lac I* ^q^, *lacZ*ΔM15: Tn*10*(*tet* ^r^)]	Clontech
S17-1	*thi pro hsdR recA; chromosomal RP4; Tra* ^*+*^ *; Tmp* ^r^ *Str/Spc* ^r^	Simon et al. (1983)

### 2.2 Transcriptome analysis

The cells of *C. necator* strain H16 pre-cultured in LB medium were harvested by centrifugation at 4,000 × g for 10 min at 25°C and washed twice with modified basal mineral (MB) medium (Kato et al., 1996) by repeating suspension and centrifugation. The washed cells were resuspended in the fresh MB medium to obtain an optical density at 600 nm (OD_600_) of 0.02. Incubations for transcriptome analysis were performed using a sealed glass bottle (124 mL capacity) filled with 40 mL of the cell suspension at 30°C with agitation at 180 rpm. For autotrophic condition, the gas phase was replaced with a mixture of H_2_:O_2_:CO_2_ (80:10:10 [v/v]) at approximately 1 atm (H_2_/CO_2_ culture). For heterotrophic conditions, the gas phase was replaced with a mixture of N_2_:O_2_:CO_2_ (80:10:10 [v/v]) at approximately 1 atm, and the medium was supplemented with 1/100 volume of filter-sterilized stock solutions of sodium acetate (2 M) or d-fructose (1 M) (acetate and fructose cultures, respectively). After 22 h of incubation (OD_600_ of approximately 0.15, 0.20, and 0.55 for the H_2_/CO_2_, acetate, and fructose cultures, respectively), the gas phase was replaced with the fresh gas mixture with the same composition, and an additional 1/100 volume of the substrate stock solutions was supplemented. The cells were then incubated for an additional 3 h under the same conditions before being subjected to transcriptome analysis. The transcriptome analysis was conducted with three biological replicates. Total RNA was isolated using ISOGEN II reagent (Nippon Gene, Tokyo, Japan) combined with a bead-beating method, as previously described (Kato et al., 2014). RNA purification using an RNeasy Mini kit (Qiagen, Hilden, Germany) with a DNase treatment and quantification by using the Qubit 2.0 fluorometer (Thermo Fisher Scientific, Waltham, MA, United States) were carried out as described previously (Xie et al., 2023). RNA samples were pre-treated as described previously (Huang et al., 2025) and sequenced by using DNBSEQ-G400 sequencer under DNBSEQ-G400RS High-throughput Sequencing Set at 2 × 200 bp model by Bioengineering Lab (Kanagawa, Japan). The raw reads were trimmed and cleaned by Trimmomatic v0.39 (phred33, ILLUMINACLIP: 2:30:10, LEADING:3, TRAILING:3, SLIDINGWINDOW:6:30 MINLEN:33, and other parameters by default) (Bolger et al., 2014) and then mapped to the genome of *C. necator* strain H16 (GCA_000009285.2) using BWA v0.7.17 (with mem algorithm, and other parameters by default) (Li and Durbin, 2009). Gene expression levels of 6,999 open reading frames (ORFs) were calculated as transcripts per million (TPM) using StringTie v2.2.1 (with -e and -G options, and other parameters by default) (Pertea et al., 2016).

### 2.3 A promoter evaluation system based on β-galactosidase activity measurements

The plasmids and primers used in this study are listed in Table 2 and Supplementary Table S1, respectively. The strategy for construction of the promoter evaluation vectors is illustrated in Supplementary Figure S1. The sequence of the *rrnB* terminator of *E*. *coli* (T*rrnB*) was PCR amplified with NsiI and XbaI recognition sequences at the 5′- and 3′-ends, respectively. The sequence of β-galactosidase gene originated from *E. coli* was amplified with XbaI-NdeI and AgeI recognition sequences at the 5′- and 3′-ends, respectively. The two PCR products were inserted at the NsiI-AgeI site of the broad host range vector pBBR1MCS-2 by In-Fusion cloning (In-Fusion HD Cloning Kit, TaKaRa Bio, Kusatsu, Japan). The initiation codon ATG of the β-galactosidase gene was constructed to overlap with the ATG of the introduced NdeI recognition sequence. The resultant vector was designated as pBBR-bgal. To evaluate the promoter activities, each candidate promoter sequence was introduced into the XbaI-NdeI site of the pBBR-bgal and the resultant vectors were designated as pBBR-xxxx as listed in Table 2. These vectors were introduced into the *C. necator* strain H16 by transconjugation using *E. coli* S17-1 as the donor (Simon et al., 1983), followed by selection of the transconjugants on Simmons Citrate Agar medium as previously described (Fukui and Doi, 1997; Mifune et al., 2010). The crude enzyme solutions were prepared from the *C. necator* strains cultured until the mid-exponential phases under autotrophic (the H_2_/CO_2_ culture) and heterotrophic (the fructose culture) conditions. Cell disruption for crude enzyme preparation was performed by beads-beating for 60 s at 2,500 rpm at 4°C using Multi-beads Shocker MB1448 (Yasui-Kikai, Osaka, Japan) with Lysing Matrix B (Funakoshi, Tokyo, Japan) in phosphate buffered saline. Protein quantification was conducted using Qubit Fluorometer (Invitrogen), according to the manufacturer’s instruction. The promoter activities were evaluated by measuring β-galactosidase activities in the crude enzyme solutions using β-Galactosidase Enzyme Assay System with Reporter Lysis Buffer (Promega, Madison, WI, United States), according to the manufacturers’ instruction. The assay was conducted with three biological replicates, and the Student’s t-test was used for the statistical analyses.

**TABLE 2:** Plasmid used in this study.

Plasmid	Description	Reference
pBBR1MCS-2	Broad host range plasmid; *mob*, *P*_*lac*_, lacZα, *kmr*, replicable in strain H16	Kovach et al. (1995)
pBBR-bgal	Derivative of pBBR1-MCS2, promoter-probe vector, containing *E. coli TrrnB* and *β-gal* (promoterless)	This study
pBBR-PS01	Derivative of pBBR-bgal, containing upstream region of H16_B1395	This study
pBBR-PS02	Derivative of pBBR-bgal, containing upstream region of PHG088	This study
pBBR-PS03	Derivative of pBBR-bgal, containing upstream region of H16_B0947	This study
pBBR-PS04	Derivative of pBBR-bgal, containing upstream region of h16_B1040	This study
pBBR-PS05	Derivative of pBBR-bgal, containing upstream region of PHG094	This study
pBBR-PS06	Derivative of pBBR-bgal, containing upstream region of H16_B1452	This study
pBBR-PS07	Derivative of pBBR-bgal, containing upstream region of PHG001	This study
pBBR-PS08	Derivative of pBBR-bgal, containing upstream region of H16_B2185	This study
pBBR-PS09	Derivative of pBBR-bgal, containing upstream region of H16_B1650	This study
pBBR-PS10	Derivative of pBBR-bgal, containing upstream region of PHG318	This study
pBBR-PS11	Derivative of pBBR-bgal, containing upstream region of PHG023	This study
pBBR-PS12	Derivative of pBBR-bgal, containing upstream region of H16_B0960	This study
pBBR-PC01	Derivative of pBBR-bgal, containing upstream region of H16_ A3402	This study
pBBR-PC02	Derivative of pBBR-bgal, containing upstream region of H16_ A2566	This study
pBBR-PC03	Derivative of pBBR-bgal, containing upstream region of H16_ A0482	This study
pBBR-PC04	Derivative of pBBR-bgal, containing upstream region of H16_ A3144	This study
pBBR-PC05	Derivative of pBBR-bgal, containing upstream region of H16_ A0566	This study
pBBR-PC06	Derivative of pBBR-bgal, containing upstream region of H16_ A0204	This study
pBBR-PC07	Derivative of pBBR-bgal, containing upstream region of H16_ A0511	This study
pBBR-Plac	Derivative of pBBR-bgal, containing *lac* promoter	This study
pK18mobsacB	Cloning vector, *mob*, *sacB*, *kmr*, not replicable in strain H16	Schafer et al. (1994)
pK18A0404-m266	Derivative of pK18mobsacB, containing *lox71*, *cmr*, *lox m2/66* genes and partial H16 genomic franking regions of the locus tag of H16_A0404	This study
pSK026-CreN	Derivative of pK18mobsacB, containing *cre*, *lox m2/71*, *lox 66*	This study
pSK026_Unit1A23	Derivative of pSK026-CreN, containing the engineered CO_2_ fixation pathway genes	This study

### 2.4 Genome modification

The genome-engineered *C. necator* strain DL_1A23 harboring a set of genes related to CO_2_ fixation were constructed as follows. Based on the RNA-Seq results, genomic loci with extremely low transcription levels under both autotrophic and heterotrophic conditions were identified, and the H16_A0404 locus was selected as the insertion site for the exogenous genes. The pK18A0404-m266 vector used to introduce the *lox* sequence, the target site for Cre recombination, at the H16_A0404 locus was constructed by incorporating *lox71*, the chloramphenicol resistance gene (*cmr*), *lox m2/66* sequences, and the flanking regions of H16_A0404 into the pK18mobsacB vector (Supplementary Figure S2). The pK18A0404-m266 vector was transferred into the *C. necator* strain IP-015 by transconjugation from *E. coli*. A double-crossover homologous recombinant strain, named IP015DL, was obtained by selection based on resistance to chloramphenicol and sucrose. The Cre recombination vector pSK026-CreN was constructed by incorporating Cre recombinase gene (*cre*), *lox m2/71*, and *lox66* sequences into the pK18mobsacB vector. The seven genes for an engineered CO_2_ fixation pathway (Supplementary Table S2) were cloned into the pSK026-CreN vector at the position between the two *lox* sites, and the resulting vector was named pSK026_Unit1A23 (Supplementary Figure S3). The seven genes were arranged in the order shown in Supplementary Table S2. The autotrophic-specific promoters PS01, PS07, and PS11 were inserted upstream of *ccr_CA*, *mcl*, and *lcc-pccB*, respectively, and the terminator T*rrnB* was inserted downstream of *lcc-pccB*. (Supplementary Figure S3). The pSK026_Unit1A23 vector was introduced into the *C. necator* strain IP015DL by transconjugation. The Cre recombinant strain harboring the seven exogenous genes and a kanamycin resistance gene (*kmr*) on its genome, named strain DL_1A23, was obtained by selecting colonies resistant to kanamycin and sensitive to chloramphenicol (Supplementary Figure S4).

### 2.5 Quantitative real-time RT-PCR (qRT-PCR)

*C. necator* strain DL_1A23 was streaked onto LB agar plates and incubated at 30°C for approximately 2 days, until single colonies appeared. Three independent colonies were picked and separately inoculated into 5 mL of NR medium (Fukui et al., 2014), then incubated at 30°C with agitation at 200 rpm. Cells were collected by centrifugation at an OD_600_ value of 0.5, washed three times with MB medium, and resuspended in 20 mL of MB medium supplemented with 200 nM vitamin B_12_ for autotrophic cultures. For the autotrophic condition, the gas phase was replaced with a H_2_:O_2_:CO_2_:N_2_ mixture (3:10:10:77 [v/v]) at approximately 1 atm, followed by incubation at 30°C with agitation at 200 rpm. The gas phase was replenished every 8 h with a gas mixture of identical composition. Cells used for RNA extraction were harvested at an OD_600_ of 0.5–0.6 during the logarithmic growth phase. Total RNA was extracted using the NucleoSpin^®^ RNA kit (Macherey-Nagel, Düren, Germany) according to the manufacturer’s instruction. The quality and concentration of the extracted RNA were assessed using a NanoDrop spectrophotometer (Thermo Fisher Scientific) and agarose gel electrophoresis with pre-staining. cDNA was synthesized from the total RNA using the ReverTra Ace^®^ qPCR RT Master Mix (TOYOBO, Osaka, Japan) following the manufacturer’s instruction. qRT-PCR was performed using the TB Green Premix Ex Taq™ II (Tli RNaseH Plus) (TaKaRa Bio) and the StepOnePlus qPCR system (Applied Biosystems, Waltham, MA, United States) under the following conditions: an initial denaturation step at 95°C for 30 s (Stage 1), followed by 40 cycles of denaturation at 95°C for 5 s and annealing/extension at 60°C for 30 s (Stage 2). After amplification, a melting curve analysis (Stage 3) was conducted, consisting of 95°C for 15 s, 60°C for 1 min, and a final step at 95°C for 15 s. The expression levels of three endogenous and three exogenous genes, each driven by one of the PS01, PS07, and PS11 promoters (Supplementary Table S5), were quantified with primers listed in Supplementary Table S1 and using the expression levels of a housekeeping gene (*gyrB*) as the internal control.

## 3 Results and discussion

### 3.1 Comparative transcriptome analysis of *C. necator* grown under autotrophic and heterotrophic conditions

**FIGURE 1:**
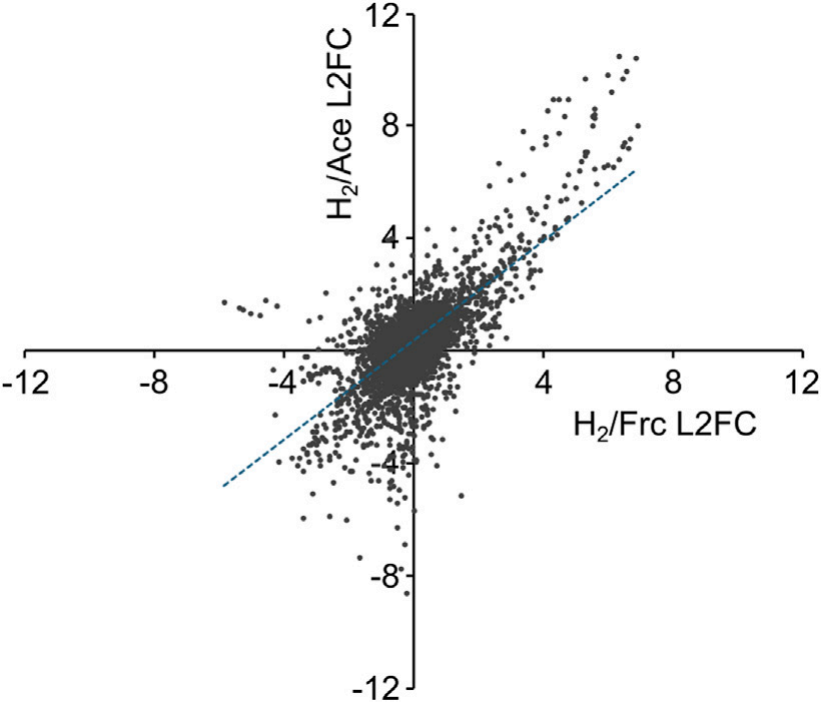
Expression profiles of 6,999 ORFs in *C. necator* H16. The log2 fold change (L2FC) values between the normalized expression values in the H_2_/CO_2_ culture (TPM-H_2_) and those in the fructose or acetate cultures (TPM-Frc or TPM-Ace) are plotted. An approximation curve (y = 0.89x + 0.32, r = 0.68) derived from the least-square method is presented as a blue broken line.

A comprehensive gene expression analysis was conducted to identify genes specifically upregulated under autotrophic conditions and those constitutively expressed under both autotrophic and heterotrophic conditions. *C*. *necator* strain H16 was cultured under autotrophic (using H_2_/CO_2_ as growth substrates) and heterotrophic (using fructose or acetate as a growth substrate) conditions and subjected to RNA-seq analysis to quantify the expression levels of each ORF. The plots of the log2 fold change (L2FC) values between the normalized expression values in the H_2_/CO_2_ culture (TPM-H_2_) and those in the fructose or acetate cultures (TPM-Frc or TPM-Ace) exhibit positive correlations, particularly for ORFs upregulated in the H_2_/CO_2_ culture (Figure 1). There were 104 and 119 ORFs significantly upregulated in the H_2_/CO_2_ culture compared to the fructose and acetate cultures (L2FC > 2, *p* < 0.01, and TPM-H_2_ > 100), respectively, among which 91 ORFs were common.

Several studies have reported comparative transcriptomic and proteomic analyses of *C*. *necator* under autotrophic and heterotrophic growth conditions (Kohlmann et al., 2011; Serna-García et al., 2024). These studies have reported that genes associated with carbon fixation and H_2_ oxidation are significantly upregulated under autotrophic conditions. In the transcriptomic analysis conducted in this study, we observed pronounced upregulation of two gene clusters encoding the CBB cycle enzymes (*cbb* operons, H16_B1395–1383 [H_2_/Frc L2FC of 3.3–6.8] and PHG427–416 [H_2_/Frc L2FC of 3.7–6.5]), as well as two gene clusters encoding hydrogenases and associated proteins (PHG001–022 [H_2_/Frc L2FC of 1.1–5.1] and PHG088–093 [H_2_/Frc L2FC of 5.6–6.9]) in the H_2_/CO_2_ culture (Supplementary Table S3). Additionally, the ORF PHG023 (H_2_/Frc L2FC of 3.5), encoding a high-affinity permease of nickel ions, essential cofactors of hydrogenases, as well as ORFs responsible for their incorporation into the enzyme complexes (PHG094–096 [H_2_/Frc L2FC of 2.1–6.0]), were also found to be highly expressed in the H_2_/CO_2_ culture (Supplementary Table S3). Previous studies have shown that expression of genes involved in C1 metabolism and the respiratory electron transport chain is modulated under autotrophic conditions, likely reflecting shifts in cellular energy status (Kohlmann et al., 2011; Serna-García et al., 2024). With regard to C1 metabolism, our data revealed elevated expression of the gene cluster encoding formate dehydrogenase (H16_B1452–1455 [H_2_/Frc L2FC of 3.0–4.8]), along with the gene for specialized elongation factor required for incorporation of selenocysteine (H16_B0947 [H_2_/Frc L2FC of 5.1], Supplementary Table S3), an essential amino acid in formate dehydrogenase (Baron et al., 1993; Atkins and Gesteland, 2000). Furthermore, the gene cluster H16_B2185–2182, encoding an efflux transporter of copper ion, necessary for the function of respiratory chain proteins, was also highly expressed under H_2_/CO_2_ conditions (L2FC of 2.9–3.9, Supplementary Table S3). In addition to these characterized genes, several genes of unknown function were also upregulated in the H_2_/CO_2_ culture. The upstream regulatory regions of these genes represent promising candidates for the development of the autotroph-specific promoters.

On the other hand, ORFs in a cluster for fructose catabolism (a putative transporter and glycolysis enzymes) were significantly downregulated in the H_2_/CO_2_ culture compared to the fructose culture (H16_B1498–1503 [H_2_/Frc L2FC of −4.2 to −5.8]) (Supplementary Table S3). Similarly, the genes encoding acetyl-CoA synthetase, which is crucial for acetate catabolism, were exclusively expressed in the acetate culture (H16_A2525 [H_2_/ace L2FC of −2.6] and H16_B0834 [H_2_/ace L2FC of −2.4]) (Supplementary Table S3). These observations are consistent with previous reports (Denger et al., 2011; Kohlmann et al., 2011; Serna-García et al., 2024) and suggest the validity of the transcriptome analysis performed in this study.

### 3.2 Selection of candidate promoters

**FIGURE 2:**
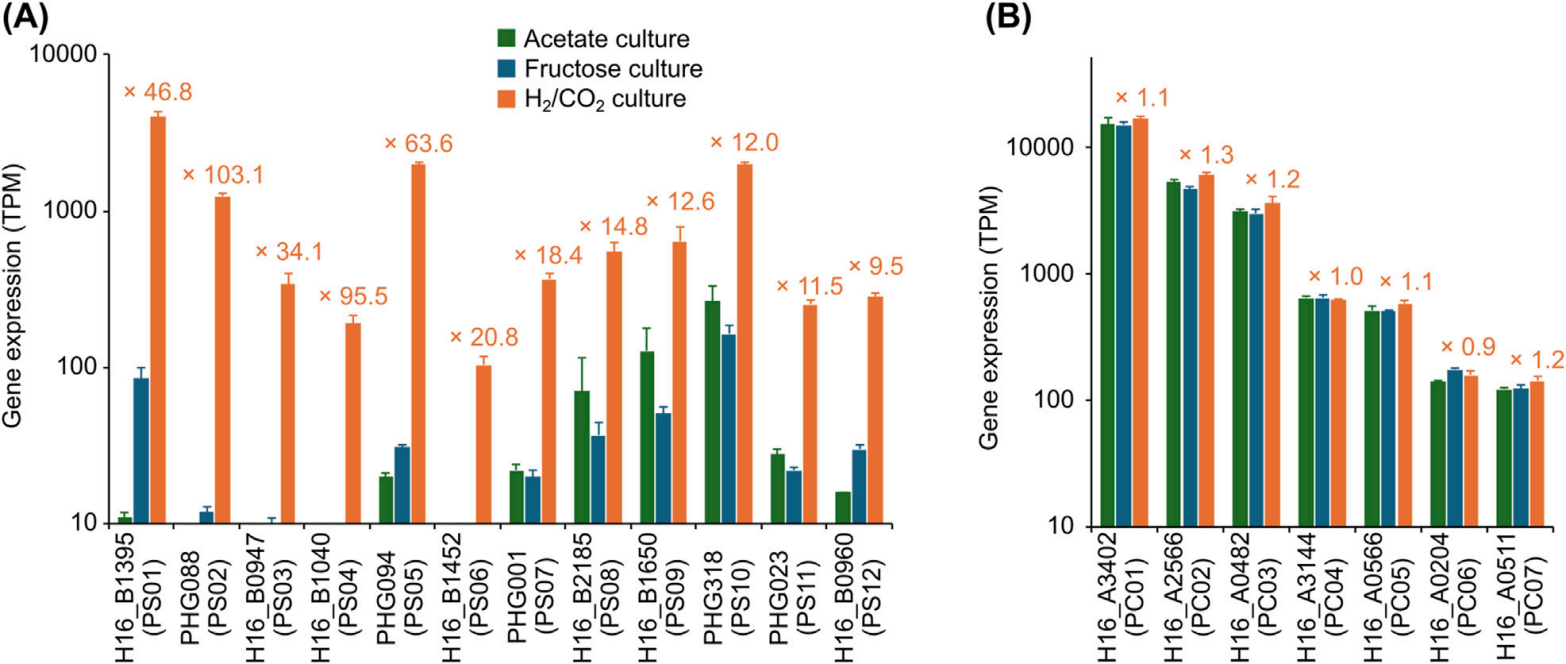
The expression data for genes used to identify candidate promoters. **(A)** Genes associated with autotroph-specific promoter candidates (PS01–PS11), and **(B)** genes associated with constitutive promoter candidates (PC01–PC07). Transcriptomic profiling was performed using logarithmically growing cells cultured on acetate, fructose, or H_2_/CO_2_, and gene expression levels are presented as normalized transcript counts (transcripts per million, TPM). For candidate promoters located upstream of operons, only the expression level of the first gene in the operon is shown. The annotated function of each gene is as follows; H16_B1395 (PS01): ribulose bisphosphate carboxylase large chain (*cbbL2*), PHG088 (PS02): NAD-reducing hydrogenase diaphorase moiety large subunit (*hoxF*), H16_B1395 (PS03): selenocysteine-specific protein translation elongation factor (*selB*), H16_B1040 (PS04): probable extra-cytoplasmic solute receptor, PHG094 (PS05): hydrogenase nickel incorporation protein (*hypA*), H16_B2185 (PS06): formate dehydrogenase alpha subunit (*fdoG*), PHG001 (PS07): membrane-bound [NiFe] hydrogenase small subunit (*hoxK*), H16_B2185 (PS07): copper resistance protein A, multi-copper oxidase (*copA*), H16_B1650 (PS09) and PHG318 (PS10): hypothetical proteins, PHG023 (PS11): high-affinity nickel permease (*hoxN1*), H16_B0960 (PS12): predicted ATPase, nucleotide-binding protein Mrp, H16_A3402 (PC01): outer membrane protein (porin), H16_A2566 (PC02): acyl carrier protein (*acpP*), H16_A0482 (PC03): LSU ribosomal protein L13 (*rplM*), H16_A3144 (PC04): LysR-family transcriptional regulator (*phcA*), H16_A0566 (PC05): phosphoglycerate kinase (*pgk*), H16_A0204 (PC06): hypothetical protein, and H16_A0511 (PC07): organic solvent tolerance protein (*ostA*). Data are presented as the means of three independent cultures, and error bars represent standard deviations. The values in orange letters above each bar indicate the fold change in the TPM values of the H_2_/CO_2_ vs. fructose cultures.

Based on the transcriptome analysis, genes specifically upregulated under autotrophic conditions and those constitutively expressed under both autotrophic and heterotrophic conditions were selected, and their promoter regions were identified. For genes specifically upregulated under autotrophic conditions, the top six genes with the highest H_2_/Frc L2FC values were selected from those with TPM-H_2_ > 100. Additionally, the top six genes (excluding the previously selected genes) with the highest TPM-H_2_ values were selected from those with H_2_/Frc L2FC > 3. In these processes, when the selected gene was part of a putative operon, the first gene in that operon was selected as the candidate gene. The upstream regions (regions without ORFs, located between the selected gene and the upstream gene) of the 12 genes (Figure 2A) were identified as candidates for autotrophic-specific promoters (PS01–PS12, the sequences are presented in Supplementary Table S4). It should be noted that *C. necator* H16 has two *cbb* operons containing genes for the CBB cycle, one on the chromosome and another on the megaplasmid, which have almost identical sequences. While both operons are specifically upregulated under autotrophic conditions, only the chromosomal *cbb* operon was targeted as the candidate promoter in this study (PS01). For genes constitutively expressed under both autotrophic and heterotrophic conditions, seven genes (Figure 2B) with various TPM-H_2_ values were selected from those with H_2_/Frc L2FC values of 0 ± 0.3. The promoter regions were identified using the same procedure as for the autotrophic-specific promoters, resulting in seven candidates for constitutive promoters (PC01–PC07, the sequences are presented in Supplementary Table S4).

### 3.3 Development of a promoter evaluation system based on β-galactosidase activity

**FIGURE 3:**
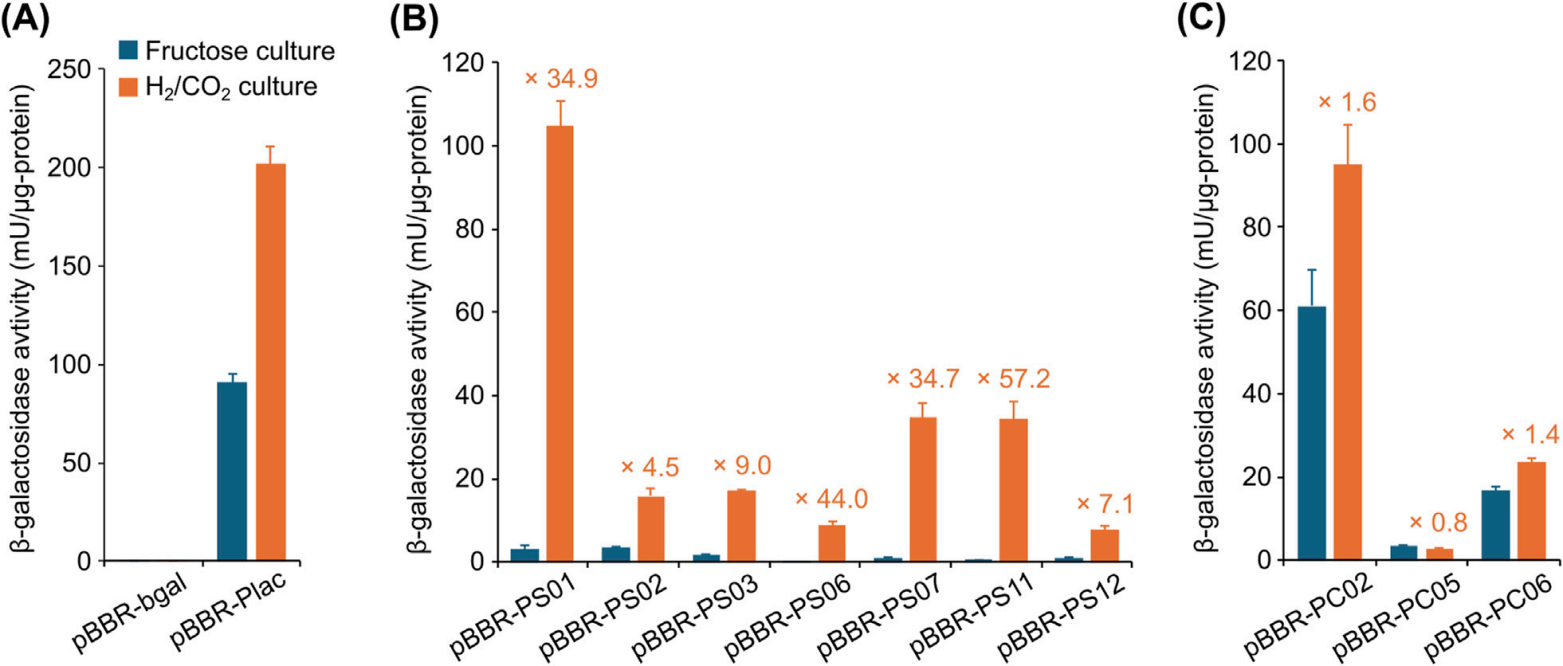
Evaluation of promoter activities by the β-galactosidase assay. β-galactosidase activities were determined using crude enzyme solutions prepared from *C*. *necator* strains harboring the indicated vectors, cultured under heterotrophic (fructose culture, blue bars) and autotrophic (H_2_/CO_2_ culture, orange bars) conditions. Each graph represents the β-galactosidase activities obtained from **(A)** the negative control strain harboring pBBR-bgal (with a promoterless β-galactosidase) and the positive control strain harboring pBBR-Plac (with the *E. coli lac* promoter), **(B)** the strains harboring vectors with the autotrophic-specific promoters, and **(C)** the strains harboring vectors with the constitutive promoters. Data are presented as the means of three independent cultures, and error bars represent standard deviations. The values in orange letters above each bar indicate the ratio of β-galactosidase activities under autotrophic to heterotrophic conditions.

Since promoter regions can influence translation efficiency in addition to transcriptional efficiency, discrepancies between mRNA and protein expression levels are commonly observed (Buccitelli and Selbach, 2020). Therefore, quantitative comparisons should be made at the protein (or enzymatic activity) levels to accurately evaluate promoters. The vector for promoter evaluation was constructed by introducing a promoterless β-galactosidase gene into the broad-host-range vector pBBR1MCS-2 (summarized in Supplementary Figure S1). To prevent read-through transcription potentially caused by the expression of upstream genes on the vector, the *rrnB* terminator sequence from *E. coli* (T*rrnB*) was introduced upstream of the β-galactosidase gene. The resulting vector pBBR-bgal was used as the promoterless negative control, and pBBR-Plac, which contains the *E. coli lac* promoter upstream of the β-galactosidase gene, was used as the positive control. The crude enzyme solutions were prepared from *C*. *necator* strains carrying these vectors and subjected to the β-galactosidase activity measurements (Figure 3A). Given the similarity in gene expression patterns between the fructose and acetate cultures (Figure 2; Supplementary Table S3), only the fructose culture was subsequently utilized as the heterotrophic condition. The crude enzyme solutions obtained from the strain harboring pBBR-Plac cultured under both autotrophic and heterotrophic conditions exhibited significant β-galactosidase activities (91–202 mU/µg-protein), while those from the strain carrying the control vector pBBR-bgal exhibited negligible levels of activities (<0.6 mU/µg-protein). These results demonstrated the validity of the promoter evaluation system constructed in this study.

### 3.4 Evaluation of the candidate promoters by the β-galactosidase assay

The sequences of 19 candidate promoters (PS01–PS12 and PC01–PC07) were introduced into the promoter evaluation vector (Table 2), which were subsequently introduced into *C*. *necator* strain H16. Among these vectors, the pBBR-PC03 did not yield any transformants despite repeated trials and was therefore excluded from subsequent experiments. Although the reason for the inability to obtain the pBBR-PC03 transformant is not clear, it may be due to the toxicity resulting from high levels of β-galactosidase expression or the inhibition of normal colony formation caused by the energy consumption associated with the constitutive expression of the enzyme at high levels. The crude enzyme solutions were prepared from the transformants cultured under autotrophic or heterotrophic conditions, and their promoter activities were assessed by measuring the β-galactosidase activities. The PS04, PS05, PS10, PC01, PC04, and PC07 promoters were excluded from the candidates because the transformants carrying the vectors with the respective promoters exhibited only negligible levels of β-galactosidase activities (<1.1 mU/µg-protein) under all the culture conditions tested. In addition, two autotrophic-specific promoter candidates (PS08 and PS09) were also excluded because the transformants carrying pBBR-PS08 and -PS09 exhibited high β-galactosidase activities under heterotrophic conditions that were comparable to those under autotrophic conditions (data not shown). Although the underlying causes for these unexpected results remain elusive, potential explanations include transcriptional regulation by unidentified elements located outside the selected sequence region, such as regulatory sequences at distant sites, and alterations in translation efficiency resulting from changes in the higher-order structure of the mRNA (Saito et al., 2019).

Figure 3B presents the results of β-galactosidase assays for the seven transformants carrying the vectors with promoters confirmed to be autotrophic-specific. The transformant carrying the vector with the PS01 promoter, the upstream sequence of the chromosomal *cbb* operon, exhibited high β-galactosidase activity under the autotrophic condition (104.8 ± 5.9 mU/µg-protein), which was comparable to that observed with P_*lac*_. Conversely, the enzyme activity was significantly lower when cultured under the heterotrophic condition (3.0 ± 1.2 mU/µg-protein), leading to 34.9-fold difference. These results demonstrated that the PS01 promoter can be used to specifically and strongly express target gene(s) under autotrophic conditions. Similarly, the transformants harboring pBBR-PS07 or pBBR-PS11 exhibited low β-galactosidase activities under the heterotrophic condition, while they showed moderate activity levels under the autotrophic condition (34.7 and 34.3 mU/µg-protein, respectively), corresponding to 34.7- and 57.2-fold upregulations, respectively. In addition, although the β-galactosidase activities of the transformants carrying pBBR-PS02, pBBR-PS03, pBBR-PS06, or pBBR-PS12 were not as high under the autotrophic condition (7.8–17.1 mU/µg-protein), they were significantly higher than those under the heterotrophic condition (4.5- to 44.0-fold differences). Figure 3C shows the results of β-galactosidase assays for the three transformants carrying vectors with constitutive promoters. The transformants carrying pBBR-PC02, pBBR-PC05, or pBBR-PC06 under the autotrophic condition exhibited similar activities to those under the heterotrophic conditions (0.8- to 1.6-fold differences), where the expression levels were distinct from each other (94.9, 2.8, or 23.6 mU/µg-protein under the autotrophic condition, respectively).

For certain promoter candidates, discrepancies were observed between transcriptomic data and β-galactosidase assay results. For example, transcriptome analysis revealed that genes downstream of the PS02 promoter were markedly upregulated under autotrophic conditions, with transcript levels exceeding a 100-fold increase compared to heterotrophic conditions (Figure 2A). In contrast, β-galactosidase activity exhibited only a moderate 4.5-fold increase between the two growth conditions. Such divergence between transcriptional and translational outputs is a well-documented phenomenon in heterologous protein expression and remains a significant challenge in the field (Buccitelli and Selbach, 2020; Pouresmaeil and Azizi-Dargahlou, 2023). Multiple factors have been implicated in reduced translational efficiency, including codon usage bias and the limited availability of specific tRNAs. Among these, the tertiary structure of mRNA is particularly influential and is likely to play a substantial role in the context of this study. Our group previously demonstrated that the tertiary structure formed by the 5′untranslated region (5′UTR), derived from the promoter, in conjunction with the RNA sequence of an exogenous gene, can impede translational efficiency (Saito et al., 2019). Moreover, we showed that modification of the exogenous gene sequence without altering the encoded amino acid can effectively disrupt inhibitory tertiary structures and significantly enhance translation (Saito et al., 2019). Further optimization of the promoters identified in this study, particularly within their 5′UTR regions, may represent a promising strategy for improving translational efficiency.

Collectively, this study successfully identified seven autotrophic-specific promoters and three constitutive promoters with distinct expression levels, which are expected to be new useful tools for development of *C. necator* strains suitable for CO_2_-based biomanufacturing. Although there have been some studies on the genetic engineering of *C. necator* to improve its function under autotrophic conditions, the promoters used in these studies were P_*lac*_ and P_BAD_, which originated from *E. coli* (Thorbecke et al., 2021; Kim et al., 2022). The *E. coli* P_*lac*_ has been reported to act as a strong constitutive promoter in *C. necator* (Fukui et al., 2011). However, high expression of genes required for biomanufacturing often give negative impact on the growth and viability of the host cells due to some metabolic burden or toxicity. Therefore, it has been reported that inducible gene expression system, functional during the bioproduction phases but not during the growth phases, can improve the efficiency of biomanufacturing (Raj et al., 2020; De Baets et al., 2024). Although the *E. coli* P_BAD_ promoter enables inducible gene expression in *C. necator* with the supplementation of arabinose (Fukui et al., 2011; Nangle et al., 2020), the addition of chemicals for induction is undesirable in practical biomanufacturing processes. Furthermore, fine-tuning the expression levels of multiple genes in metabolic pathways has been shown to be beneficial for efficient bioproduction (Jung et al., 2021; Ding and Liu, 2024). The promoter library developed in this study will be an effective tool to meet these demands, i.e., fine-tuning of gene expression without the need for specific external inducers, and is expected to accelerate CO_2_-based biomanufacturing and support the development of sustainable bioprocesses. Furthermore, based on the information obtained in this study, the promoter library will be further enriched through optimization of the promoter region and length, as well as improvements via promoter engineering (random mutagenesis, hybrid construction, etc.) (Johnson et al., 2018; Cazier and Blazeck, 2021).

### 3.5 Expression of exogenous genes introduced into the *C. necator* genome by the autotrophic-specific promoters

**FIGURE 4:**
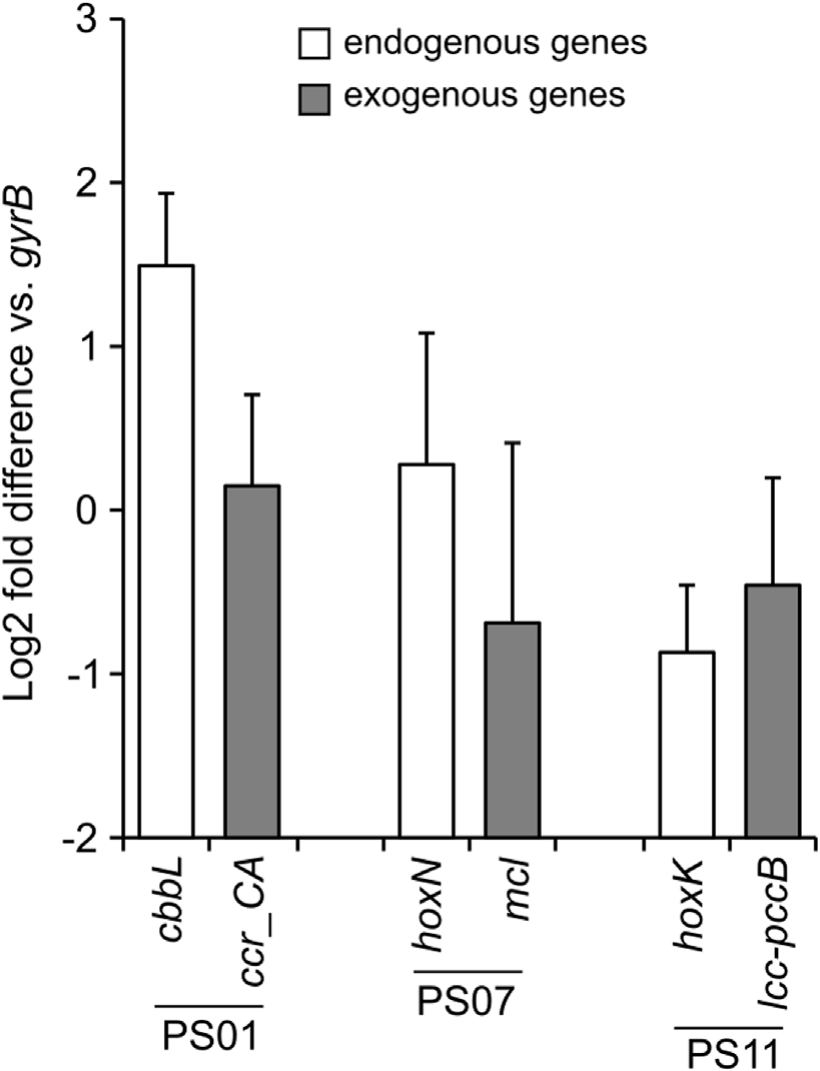
The expression levels of exogenous and endogenous genes in the genome-engineered *C. necator* strain DL_1A23. The expression of three exogenous genes (gray bars) introduced into the *C. necator* genome and three endogenous genes (white bars) located downstream of the corresponding original promoter regions (PS01, PS07, and PS11) under autotrophic conditions was determined by qRT-PCR analysis. Expression levels are represented as log2 fold differences relative to the housekeeping gene *gyrB*. Data represent the means of three biological replicates, with error bars indicating standard deviations.

To evaluate the ability of the autotrophic-specific promoters identified in this study to regulate gene expression within a genomic context, we constructed a genome-engineered *C. necator* strain DL_1A23. Although this strain was constructed to enhance the CO_2_-fixing ability by introducing seven exogenous genes (Supplementary Table S2), the functions of each gene and the characteristics of the strain are beyond the scope of this study and therefore will be discussed elsewhere. The seven exogenous genes were integrated into *C. necator* chromosome 1 via Cre/Lox recombination (Supplementary Figure S4). In this strain, three autotrophic-specific promoters were employed: the promoter of the RuBisCO large subunit gene *cbbL* (PS01), that of the hydrogenase gene *hoxK* (PS07), and that of the permease gene *hoxN* (PS11). These promoters were inserted upstream of the exogenous genes, *ccr-CA*, *mcl*, and *lcc-pccB*, respectively. *C. necator* strain DL_1A23 was cultured under the autotrophic conditions, and the expression levels of the three exogenous genes, as well as the three endogenous genes downstream of respective original promoter regions, were evaluated by qRT-PCR (Figure 4). The expression levels of the three endogenous genes were comparable to that of the housekeeping gene *gyrB* (0.5 to 2.8-folds), following the trend *cbbL* > *hoxN* > *hoxK*, consistent with the results of the RNA-seq analysis (Figure 2A). The expression levels of the three exogenous genes were comparable to those of the endogenous genes sharing the same promoter regions (0.4 to 1.3-folds), demonstrating that the promoters identified in this study can function effectively within a genomic context. Although the differences were not significant, the downstream genes of the PS01 and PS07 promoters (*cbbL* and *hoxN*) tended to exhibit lower expression levels, while the downstream gene of the PS11 promoter (*hoxK*) tended to exhibit higher expression levels compared to the corresponding endogenous genes. While promoter activity should ideally remain unaffected by the identity of downstream genes, it is plausible that differences in promoter length and sequence range, as well as the genomic locus of exogenous gene integration, may have influenced transcriptional efficiency. In addition, it is frequently observed that the expression levels and patterns of genes are altered when exogenous genes are introduced via plasmids or integrated into the genome (Nakamura et al., 2025). Further evaluation and improvement of the promoters identified in this study may be necessary to fine-tune the expression of exogenous genes introduced into the genome.

## 4 Conclusion

In this study, we established a novel promoter library for *C. necator* useful for biomanufacturing from CO_2_, which enables gene expression specific to autotrophic conditions. We identified seven autotrophic-specific promoters and three constitutive promoters with varying expression intensities, all functioning independently of specific external inducers, particularly when exogenous genes are introduced via plasmids. These promoters would serve as valuable tools for the practical application of *C. necator* in CO_2_-based biomanufacturing. Further research, such as promoter engineering, could enable more precise control of gene expression.

## Data Availability

The raw sequencing reads of the RNA-seq analysis have been deposited in the DDBJ Sequence Read Archive under the accession number DRR628345–DRR628353.
