# Supplementary material for: A promoter library for tuning gene expression in Cupriavidus necator under autotrophic conditions
Source: Front Bioeng Biotechnol. 2025 Jul 4;13:1595440. doi: 10.3389/fbioe.2025.1595440 (PMC12271211; doi:10.3389/fbioe.2025.1595440)
Supplement: Supplementary file 1 [file DataSheet1.pdf]

A promoter library for tuning gene expression in *Cupriavidus necator* under autotrophic conditions

Wataru Kitagawa[1,2], Kensuke Igarashi[1], Ryo Nagasawa[1], Shigeyuki Kakizawa[3], Mizuki Horino[4,5] Kosuke Fujishima[4,6], Toshiaki Fukui[7], Souichiro Kato[1,2,8*]

[1]Bioproduction Research Institute, National Institute of Advanced Industrial Science and Technology (AIST), Sapporo, Japan

[2]Division of Applied Bioscience, Graduate School of Agriculture, Hokkaido University, Sapporo, Japan

[3] Bioproduction Research Institute, National Institute of Advanced Industrial Science and Technology (AIST), Tsukuba, Ibaraki, Japan

[4] School of Life Science and Technology, Institute of Science Tokyo, Tokyo, Japan

[5] Earth-Life Science Institute, Institute of Science Tokyo, Tokyo, Japan

[6] Graduate School of Media and Governance, Keio University, Fujisawa, Japan

[7] School of Life Science and Technology, Institute of Science Tokyo, Yokohama, Kanagawa, Japan

[8] Research Center for Solar Energy Chemistry, Graduate School of Engineering Science, Osaka University, Toyonaka, Osaka, Japan

Supplementary Material

Supplementary Figures S1-S4.

Supplementary Tables S1, S2, S4 and S5.

(Supplementary Table S3 is provided as a separate file)

Supplementary Figure S1. Summary of construction of the promoter evaluation vector (pBBR-bgal).

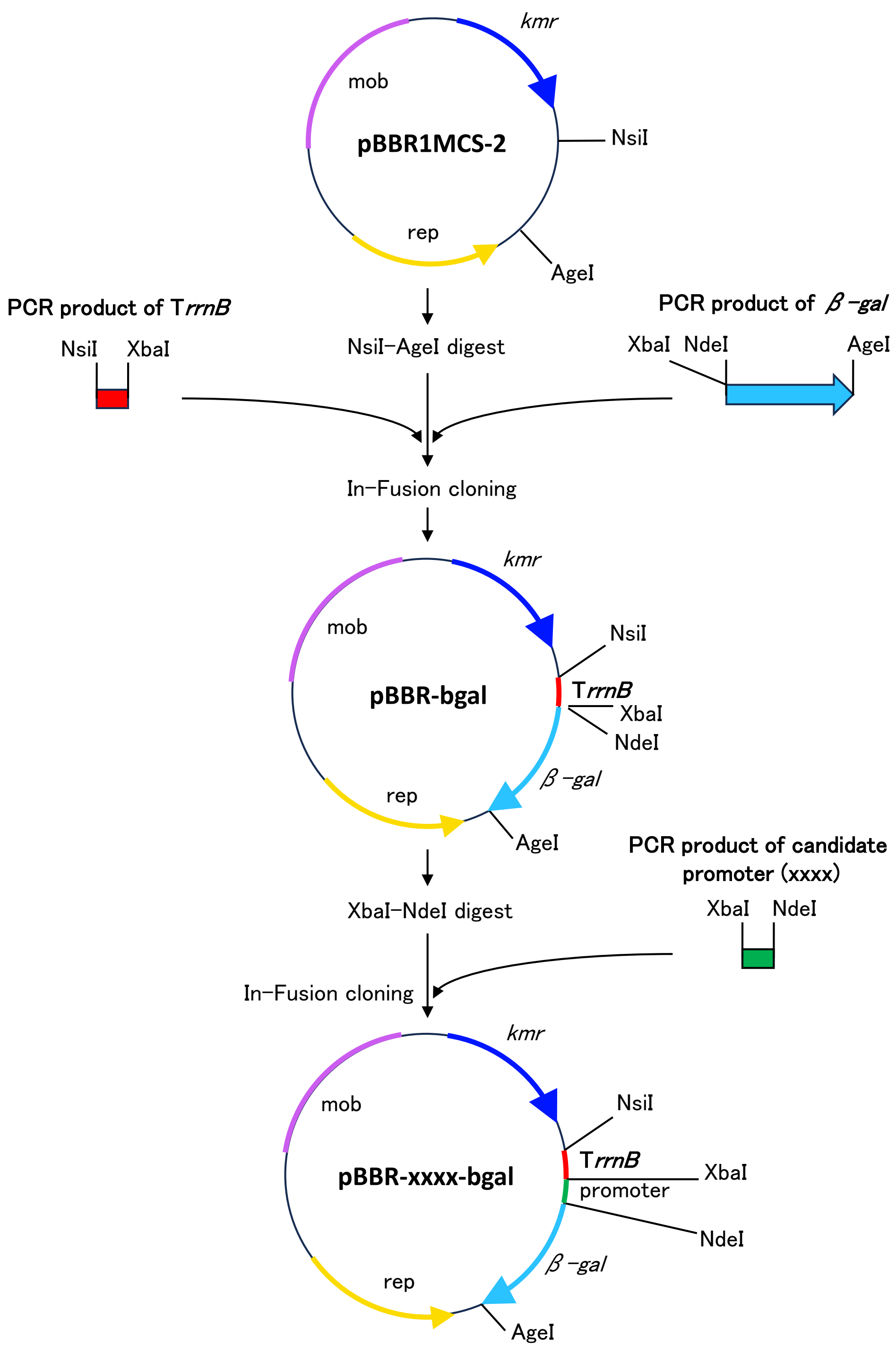

Supplementary Figure S2. Summary of construction of the pK18A0404-m266 vector.

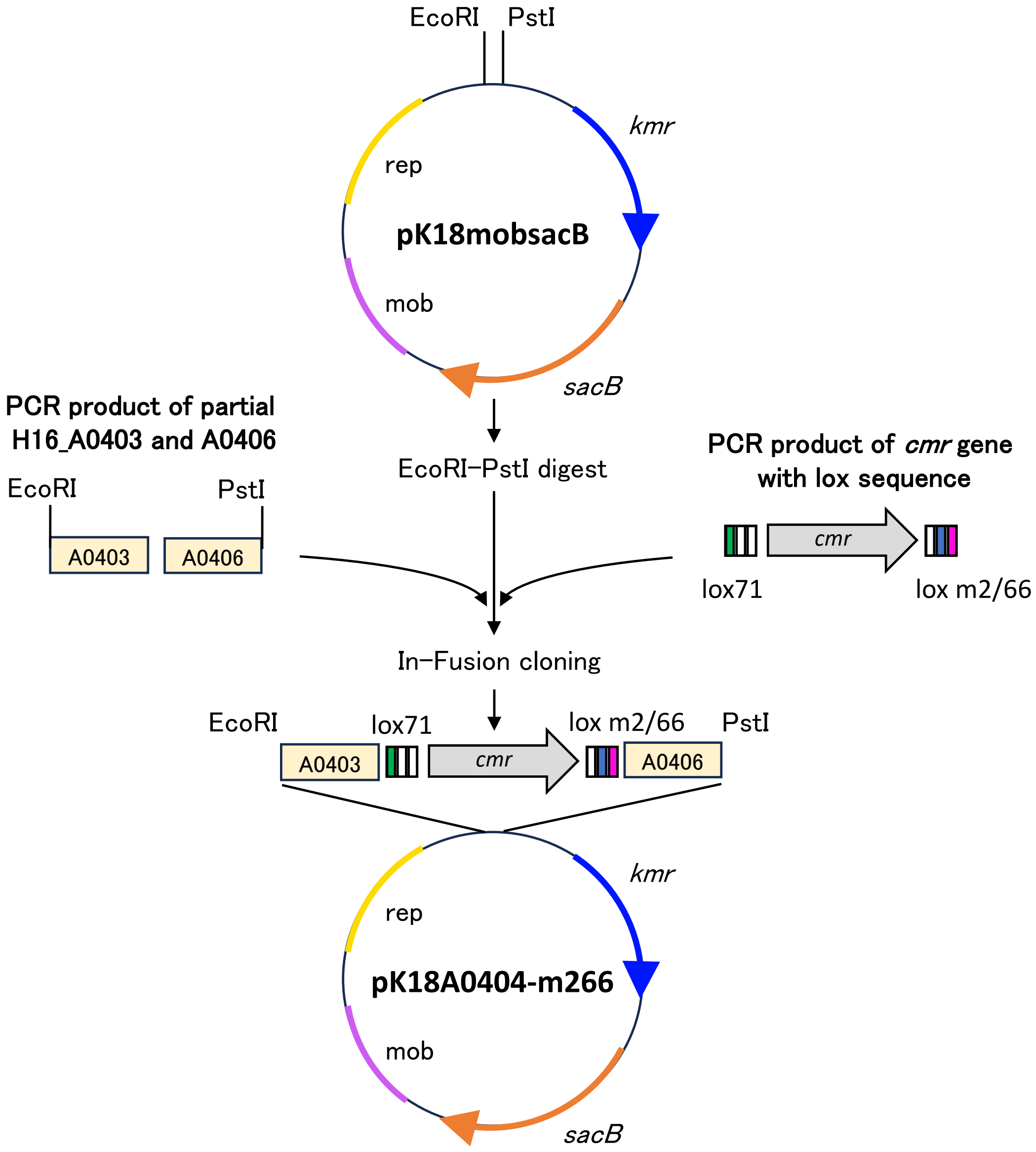

Supplementary Figure S3. Summary of construction of the pSK026_Unit1A23 vector.

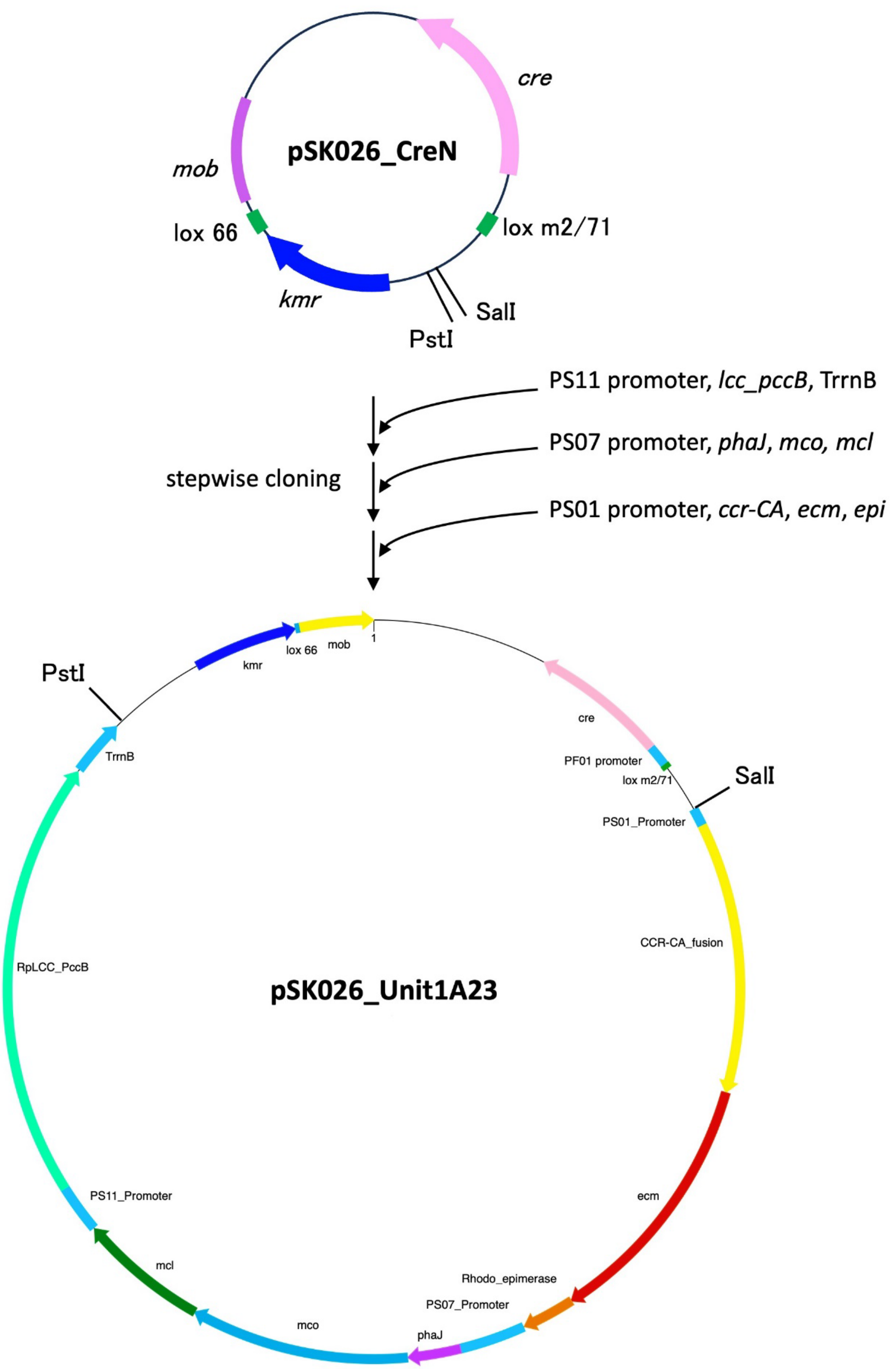

Supplementary Figure S4. Summary of construction of *C. necator* strain DL_1A23 harboring the *lox* and the engineered CO_2-fixation pathway genes.

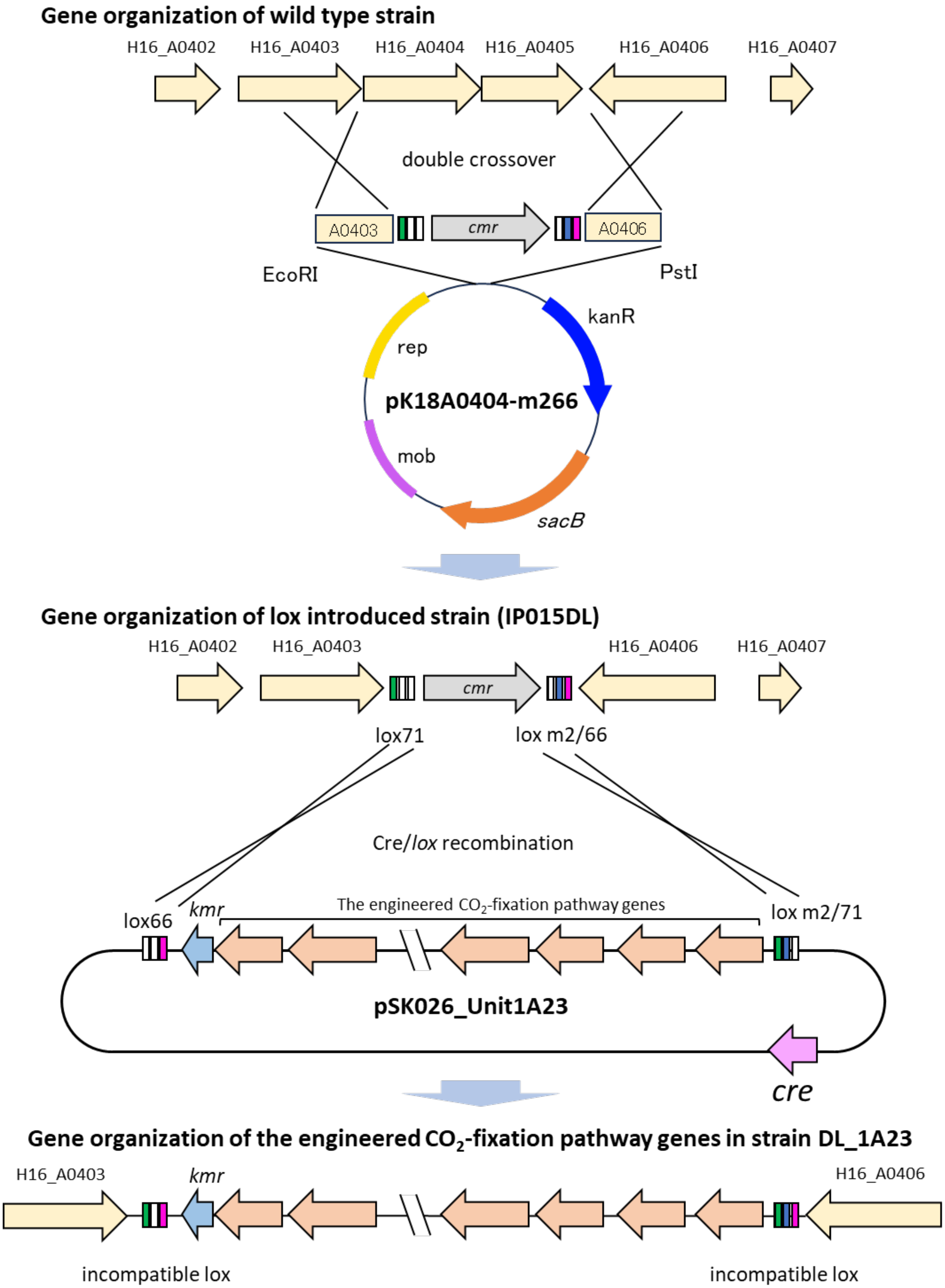

Supplementary Table S1. Primers used in this study.

Primer ID	Nucleotide sequence	Usage
TnnrB-F, -R	F: TTACAACAGTTTTTATGCATCGGAAGATCTGGATCGAAC	Construction of pBBR-bgal
	R: CATATGTAATAATCTAGATAGAACTAGTGGATCCTTGTAG	
bgal-F, -R	F: TCTAGATTATTACATATGACCATGATTACGGATTCAC	Construction of pBBR-bgal
	R: TAGTCAATAAACCGGTTTATTTTTGACACCAGACCAA	
PS01-F, -R	F: ACTAGTTCTATCTAGAGTGATGTGCAGCTTGGTC	Construction of pBBR-PS01
	R: GTAATCATGGTCATATGTGTCTCCTTGCGTGGTTG	
PS02-F, -R	F: ACTAGTTCTATCTAGAGTGACTGCCTCCGGTCAC	Construction of pBBR-PS02
	R: GTAATCATGGTCATATGGTCTCCTCCTTACTAATGTT	
PS03-F, -R	F: ACTAGTTCTATCTAGACGGACTCTTCATGTTGGTT	Construction of pBBR-PS03
	R: GTAATCATGGTCATATGTGCGTCAGGTAGCCCGGC	
PS04-F, -R	F: ACTAGTTCTATCTAGAGGTTCCGTCTCCTCCGGA	Construction of pBBR-PS04
	R: GTAATCATGGTCATATGTAAGCACCAGGCAGAGTG	
PS05-F, -R	F: ACTAGTTCTATCTAGATCACTCTCCGCGTCCAGC	Construction of pBBR-PS05
	R: GTAATCATGGTCATATGCGGCTCCTACCATGTCGG	
PS06-F, -R	F: ACTAGTTCTATCTAGAAGAAACGGCCAGCATGGA	Construction of pBBR-PS06
	R: GTAATCATGGTCATATGGGTCTCATCGTCATTTCTTCTTC	
PS07-F, -R	F: ACTAGTTCTATCTAGAGCATGCAGCAGGGGTACA	Construction of pBBR-PS07
	R: GTAATCATGGTCATATGCTGTCTCCTAATTTCTGTATTGGTATTG	
PS08-F, -R	F: ACTAGTTCTATCTAGAAGCACTTTCCTTGATCGATCG	Construction of pBBR-PS08
	R: GTAATCATGGTCATATGATCAGTCCTGGTAGAAAGCATGC	
PS09-F, -R	F: ACTAGTTCTATCTAGAGACTCTCGTGAGAGGCCC	Construction of pBBR-PS09
	R: GTAATCATGGTCATATGTCGCTTCTCCGTAAGGGT	
PS10-F, -R	F: ACTAGTTCTATCTAGAGGCGTTTCCTTGGATGTAAC	Construction of pBBR-PS10
	R: GTAATCATGGTCATATGGATCAGAAGCCTGCCACTG	
PS11-F, -R	F: ACTAGTTCTATCTAGAAACGCCTGTTCGAAATTGG	Construction of pBBR-PS11
	R: GTAATCATGGTCATATGGGAAGTCTCCTGTCTCGGTTC	
PS12-F, -R	F: ACTAGTTCTATCTAGAGGGTTGATCCGCGACGAT	Construction of pBBR-PS12
	R: GTAATCATGGTCATATGATACTCCGCTGCTGGTGAATG	
PC01-F, -R	F: ACTAGTTCTATCTAGATTCGGCTGAGGTAGGCCT	Construction of pBBR-PC01
	R: GTAATCATGGTCATATGCATTCACGTCTGCTGCGT	
PC02-F, -R	F: ACTAGTTCTATCTAGATGGGGTTTTGGCTGGCTG	Construction of pBBR-PC02
	R: GTAATCATGGTCATATGACCCTCCAGGGAAGTTCG	
PC03-F, -R	F: ACTAGTTCTATCTAGACCGCCAGTCCCTCCGTCG	Construction of pBBR-PC03
	R: GTAATCATGGTCATATGTTTTCCTGACTATTAGTTCGT	
PC04-F, -R	F: ACTAGTTCTATCTAGAGATGGCCGGGATGAGCCG	Construction of pBBR-PC04
	R: GTAATCATGGTCATATGCAGGCCTCGGTCAGATTA	
PC05-F, -R	F: ACTAGTTCTATCTAGACTGACTCACCCCCTGCAT	Construction of pBBR-PC05
	R: GTAATCATGGTCATATGGATGGATGGCGCGAGATT	
PC06-F, -R	F: ACTAGTTCTATCTAGAGGCGCGAGTGTATCAATG	Construction of pBBR-PC06
	R: GTAATCATGGTCATATGAGGCTCTCCTGAAAGCAG	

PC07-F, -R	F: ACTAGTTCTATCTAGAACAGATACGTGTGGGGAAC<br>R: GTAATCATGGTCATATGCACGGGTTGTCAGTCTCG	Construction of pBBR-PC07
Plac-F, -R	F: ACTAGTTCTATCTAGAAAATCATAAAAAATTTATTTG<br>R: GTAATCATGGTCATATGTAATTTCTCCTCTTTAATG	Construction of pBBR-Plac
Ptac-F, -R	F: ACTAGTTCTATCTAGACCCGCGGGGTTCTGGCAAATA<br>R: GTAATCATGGTCATATGAATTCTGTTTCCTGTGTGA	Construction of pBBR-Ptac
A0404-m266-F1-F, -R	F: ACATGATTACGAATTCGACACCATGGTCCGCTACTC<br>R: TAGGTTAATGCGAACAGCACTACCTGGATG	Construction of pK18A0404-m266
gyrB-qF2, -qR2	F: CAGATTGAGGATGCTATTG<br>R: TCAAACATCAATATTCCGC	Quantification of *gyrB* expression by qRT-PCR
ccr_CA-qF1, -qR1	F: GAATACGAAGACCTGTTC<br>R: GATGAGGAATTTCGGGA	Quantification of *ccr_CA* expression by qRT-PCR
mcl-qF2, -qR2	F: GACGTGATCAACCTGGAC<br>R: TTGATGGCTTCGATGATGTT	Quantification of *mcl* expression by qRT-PCR
LccPCCB-qF1, -qR1	F: GAGAAGACCGCAGAATA<br>R: GCAGGATTATATCGTCTACC	Quantification of *lcc-pccB* expression by qRT-PCR
cbbL-qF1, -qR1	F: CGACACGCCGTGGTTCTTCT<br>R: GTGCCGCCAGAACGACATGA	Quantification of *cbbL* expression by qRT-PCR
hoxK-qF1, -qR1	F: ACGACGAATCAGCCCGCAAA<br>R: TGGAGCAGGCGTTGTACGTG	Quantification of *hoxK* expression by qRT-PCR
hoxN1-qF1, -qR1	F: GTCGGTGGCATTGAGACGCT<br>R: TCGACACGACCCAACACACG	Quantification of *hoxN* expression by qRT-PCR

Supplementary Table S2. List of the engineered CO_2-fixation pathway genes used for construction of the genome-engineered *C. necator* strain DL_1A23.

Gene	Function	Origin	Reference
ccr	crotonyl-CoA carboxylase/reductase	*Methylorubrum extorquens* AM1	Insomphun et al., 2015
CA	carbonic anhydrase cadmium bound domain 2	*Conticribra weissflogii*	Alterio et al., 2015
ecm	ethylmalonyl-CoA mutase	*Rhodobacter sphaeroides*	Erb et al., 2008
epi	methylmalonyl-CoA epimerase	*Rhodobacter sphaeroides*	Erb et al., 2008
phaJ	MaoC family dehydratase	*Aeromonas caviae*	Hisano et al., 2003
mco	acyl-CoA dehydrogenase family protein	*Rhodobacter sphaeroides*	Schwander et al., 2016
mcl	L-malyl-CoA/beta-methylmalyl-CoA lyase	*Rhodobacter sphaeroides*	Erb et al., 2020
lcc	long-chain acyl-CoA carboxylase	*Rhodopseudomonas palustris*	Tran et al., 2015
pccB	acyl-CoA carboxylase subunit beta	*Bacillus subtilis*	Liu et al., 2020

Supplementary Table S4. Nucleotide sequences of promoters used in this study.

Promoter ID	Nucleotide sequence	Locus tag[a]
PS01	GTGATGTGCAGCTTGGTCCGCACTTAAGGGATTGCTTATACAGGGGCTAAGAATATCTG<br>AATTTACCTTATGTGGGTGGGCTTATATCTTTGCATCAACGCAGCAGCCAAGACGCTCA<br>ACCACGCAAGGAGACA	H16_B1395
PS02	GTGACTGCCTCCGGTCACCCGGTGCTCGGGGTGCGATTCCCCGGGTCTACTTACCAAAT<br>CGGCCGCGCACCCAATGAGAGGCGCTGGCACAAGCTTGCACAGACTTGCCCGCCAAGC<br>GGAAGCAGCCTTGCCACATCGGCCGACCCAATGGCAATGCCGCTGCCACCCGCCGGAT<br>GGCCGTTCTGGAAACGGCTTGAGCGACGTCAAGAATTTCCTTTCTCGACAAGCACTTAG<br>CCGGGCCTCCTGGTGGTTTCCCTTAGGCCCTGCGAAATTGGCGCACATCCTGCGTTCCAC<br>CTGCGCATCGAAGTGACGCACCAAGCAAGGGGCGAACATTAGTAAGGAGGAGAC	PHG088
PS03	CGGACTCTTCATGTTGGTTATACCGATTCGGTTTGCGGGCTGACGGAAGAGAATTCAAC<br>GCATAAGCCTACACGAATTGAGTGGCGCCTCTGACCTTTCATTTTCTCCTGACCAGGCA<br>GTGTCAGTTGCAGTTGTCAATCCAGGCAGCTGGAGCAAGGCCTGTGCCTGTGACGGCAT<br>CCTTGGGTAGTCAGGCGGTTCGGGCGCGCGGCAAGCGCTGCGCGGCATCCGCGACGGA<br>CAAAATGTCCATGCTGAGCCAGGTCTCGCGGGTCATTTTGTCCATTCCGTTGTCACATAA<br>GGCATCGCTTATAGGCACGCAAAGAATCGCTGAATTTACCTTAAGTGGCCAGGCAATTA<br>TCTTGTCTCCAACGATGCAGGAACACTAAGCAAAGCGGCATCGAAGCACGCTTCAGCA<br>AGCCTCATCGGCCATTCAAACAAGGATAGGGGAGACCATGCATGATCACGACGAGGAT<br>ACCCCGGAGACCATCCCTGATGGTCTCCGTCACGTCAGTCTGCCGGGCTACCTGACGCA	H16_B0947
PS04	GGTTCCGTCTCCTCCGGATAAGCGGCGCGATACCTGCATTGGGTAGCGCCTGCCCGGAG<br>CTGAGCAGGTTTCGTACCGTGCATCGGGAGTCCGGCACTTGCGCGGTGAGCGGCCCGGG<br>AACGCGCTGGCCGAACCCGCCTTGCGCCGATGCCTGTCACAAAACAGGACAGCTGCGG<br>ACACAAGCAGCGGGCCGGTGCTCAACCATGGGACAGTCGTTCGCAGCGCACCGCACAA<br>GGCACGGCCTGGCTGCAGCGTCCGGCAGTGCGTCCGGCGCGGCCAGGCAACGGCTGGC<br>ATGGAGCGCGGCCTGATGCTGCGCTGCACATGGTATCGGCCTTGCTCTGTTCACCTGCA<br>TCAACACTCTGCCTGGTGCTTA	H16_B1040
PS05	TCACTCTCCGCGTCCAGCTGAAATGCAAAAAAGCATATCCCTCGAGCGCCAGCGACGTG<br>CGTCCCGTCGAGTGGTACCTCAGCGCGTGACCGACATGGTAGGAGCCG	PHG094
PS06	AGAAACGGCCAGCATGGAAGTCAAGCGTGACGCTTCCTTGGGGCATGAGCCGCCCGCG<br>CCGAACCGGACTTTCCCCTATTTGGAGTAATCGGCCGGTCGTGCTACGCTCCTCAGCGA<br>GCGATACCCGTCCCGACGTCGTGCCCCGGTGGGGCCGCGCATCTGCAGGGCAATCCGCA<br>GGGCGTTTCGGGCTCATTCTCATCGCTTATCCAAATTAGTCTGCATTCGAACCTGAGCGG<br>CATTTCTCCGCTCGTGGAGCCGTGCGCGCGGAAAACCGGCACGGATGACGTCGCGTCGT<br>GACCGGCGCCGCGACGTCATTTGTGGTTGGTCATCCTCTTGCGCATGCACCTCAGGGAC<br>GTGCCGGACGGGGCATCTCCGTGATCGATCGCGATCCGGACGCCCCGAGGATACGTGC<br>GCCCAAATGAAGCTGCGGGTCTCGAATGCGATCGCCCCCACCCCTGGCAGCAGGGATG<br>GGCGGAAGAAGAAATGACGATGAGACC	H16_B1452

PS07	GCATGCAGCAGGGGTACAGGAAGGCTGCGAGCAGCGCCGGCTCATTGCCTTTCCGTTGG<br>GCGGGGCGAGACGCCGGGGGCGGGGGCTCATCCGAGTTCAACGCCGATCACTGAACTT<br>CCTTCTGATGCATTCAAGCGAAAACCCAGTGAGCATCTGGCGTCGGCTAGCGCCAGGCG<br>ACGGTCCACTTCATGACGGATGAAATATTGTCAAATCAGGATCCGGTGTCCTGCGTTGT<br>AGGTTCGCCGAATAGGGCGCTGTCGGGCGGACGCACGAACCTGCGTCACAGATGCTCA<br>TACATGCCTTCTCGGTATCAATCTTTTTCTAAACAAGCCATCCAACTCAGGATGGTAGCG<br>GGGGTTTTCCCCAGGTCTTCGGATTCAGGCATAGATCTTGTTTCAACTATGTCGCCAAGC<br>CAGCATTCGTGCGCGAGGGCGGTATCGCTCCCCGGTTGGCGCATCGCGACGAATGCCAA<br>TACCAATACAGAAATTAGGAGACAG	PHG001
PS08	AGCACTTTCCTTGATCGATCGGCCCTTTGGGCAGGGTCCGGCAACTCTACGCCGGCAAC<br>GCGGGCGCCAACATGACCGGAAGATTACCGCAATGTCATGTTCCGGTCAGACGTGCGTA<br>GGGTGGCCTTGGCTACGCTTGGCATGCTTTCTACCAGGACTGAT	H16_B2185
PS09	GACTCTCGTGAGAGGCCCGCTGCATTTCCGCGGGCAATGAAAAACCGGCGATGGAACC<br>GCAGGCCGGTCGGATAGCCGTGATTCTACAGCCTTTGCCCGGCGCTGCTGCGTTTCCGT<br>CGTGGGCGCGCGGCCAATTGCCGTGCGCTTGCCGCGCTCGTCCGCTGCCGAGTGACAGC<br>AATGTCATGTGCCGGTCATGCTCGTGTGGGCACGCGCTGCCTAGACTTGCTTCACCGCC<br>GCCCATTTCGGGCCGGCACCCTTACGGAGAAGCGA	H16_B1650
PS10	GGCGTTTCCTTGGATGTAACTTGGGGAAACGCCCCACCGGGGAGCGTTCCCCGGTGGGT<br>AGTGGATTGGCGGCTGACCAGGACGGTCAGCCCGCAGTGGCAGGCTTCTGATC	PHG318
PS11	AACGCCTGTTCGAAATTGGCGGAGGCAGGAGGCTGATGGCCTGATTTCCCTGCTGCACC<br>AGGCTAGAAAGCGCTGCTCCGGCTATTTAGACTCCCATGGAACATGGTATTGCCATCTG<br>GATATGGGCATGTCACCAATGCGATGATCATGCAAACCTGCTTTGCAGTCCTCACGTAC<br>GGACTTGCGCAGCAGATACCGCTATTTCGGGAATAGCATAAGCGAACCAAGACCTGAG<br>AGTGAGCTTCTGCCGCATTCGCCAGGAGTTGGCTCGCAGGCGCGGAAATTGCGTTACGG<br>TGCAGTCGAGCCTTACTGGCAAAAGCCGCGGATGACAGCGGCGTCGGAACCGAGACAG<br>GAGACTTCC	PHG023
PS12	GGGTTGATCCGCGACGATCATAGGGTAGACGACCAGCTGCCGCTGCCCACAACGCCGC<br>CCAGCGTCTCAGTAGCGGATTCTGTGGTCCCGCGATGTGCTGCTTGATCGCAACTTAAT<br>GGATTGCTTATGCGTCGGCTAAGAATATATGAATTTACCTTAACTGGGTGAGCAGATAT<br>CTTTCCAGCATTGCCGCCACGCCGCCAAAGCACGCCTGGTGCATGCATCCCTACATTCA<br>CCAGCAGCGGAGTAT	H16_B0960
PC01	TTCGGCTGAGGTAGGCCTTTCGTATCTGCTAATCCCCGTTTTTGCACCAAAACGGGGATT<br>TTCCCTCCCATTCCTGTTGCTCCCCCACAACAAAAACCGCCAGTAAGCATGGTTTACGCC<br>GAAAAAGCCTTCTGACGGCCGGGGGGGCTGGCACATAATACGCAGCAGACGTGAATG	H16_A3402
PC02	TGGGGTTTTGGCTGGCTGGTTTCGGGGGAGAAGCCCGAAGAAGCCGCCTGAAGTAGTA<br>GTTTTCCGAACCAAACGGGCGCGATTCTTGCAAAGCTTGGCAGGCGGCATTTCTGTCGC<br>CTCAAACCTGCTAAAATGCGCCCACTTTTTGTCGAACTTCCCTGGAGGGT	H16_A2566
PC03	CCGCCAGTCCCTCCGTCGATCTCCCAGTGTCTCCTCCACCCTCCTCCTTTGGTGGATTCA<br>AACCCAAGCTCAACCGCTTGGGTTTTTTTTCGCCCCTGCGTTCTGGCGCGCTAAGCACTG<br>AAAAGCGCGCGACGCTGCCCTGGTTTGGGCCCGCGATGGCGGCGACCTTGACGAAAAG<br>GTCAGTCTTTGCTTGTGCGGTTCCGGTAGCCGCCAGTATAATGGAAGGCTTTCCGTCATA<br>CCCGCGGAAGAAAGAGCAGGATCAGGCCAGCCCTCACGGGCCAGCCACAAGACTTGCA<br>GGGGCGAGTACAAGCCTTCGCGAGTCGGATTTCGGGTACGAACTAATAGTCAGGAAAA	H16_A0482

PC04	GATGGCCGGGATGAGCCGTCAGAAAAGAATGATAAAAATGGGAACGGCGGACCCACTA<br>TACCCGGATGTACGAGTGCATGTTGCGGCGCGGGAAATGTTCACATATGCGGTCAATTG<br>TGGAAAAAGAGCGCAATTTTTCAGAAATATGGCGTAGACGGCCATTTCAGAAATGCCG<br>AATTTGCTTTCCGAGCTTGTTTTTTCCTCTTACACTATTAAGACGCCGTTGAAATCTGAT<br>GTGCAGCCAGTGCAAGTGGTGGGGCCATCTAGCTAAGAATAATCTGACCGAGGCCTG	H16_A3144
PC05	CTGACTCACCCCCTGCATTCGCGACAAGGAAATGCGCGGCGCCGGCGCTGCGTCTGATC<br>GCACACCGGCTGCATTCTTTTTGGCTGCTTGTTATTTTGGCCGCCTGTTGTCATGGCCGTT<br>TCAAGACCGGCTCTGGCGCTTTTTGCGTGTCTGTTATAGCAGACGTTGCATACATTACCG<br>CACTCGGGGGTCAAGAGGGGCATGGAGCTGACCCTTCTTTCGTGGGGAGGTATGTCACT<br>AGCACCCTGTGGGGTTGTCCTGAATGTGCTGCAAGGGCCGCATGCTAAGTGCGTAACGG<br>TCCGGAATCGGCAAAGCGTTGACCACGATCAGGTAAAATGCCCGTTTCCCCGAATTCCA<br>GCGAAATCTCGCGCCATCCATC	H16_A0566
PC06	GGCGCGAGTGTATCAATGCGGCCGCGGGGCGCCGGTGCGTGCGACACCTTGCCGCATTG<br>GCGCCGAGCTTGTCGGGCTGCCAGACTAGCCGGCTGTGCGCCGCATGGCGGGGTTGCCT<br>GGGGCATGCACGGGCGGGCATCGCCATGGTCTAATGTCTGCTTTCAGGAGAGCCT	H16_A0204
PC07	ACAGATACGTGTGGGGAACGGGCGCAGAGTGGAAACGTAAATGAAACCGAAAGTAAG<br>CGGGAAGGCGACCCTAGGTTGCCCCGTATAATACCCGACCAGACCCGGGCGCCGGGCG<br>GCCCCCGGGTGGGGCCATTCGGTGCCCCGCAGCCTGCTCCGGCGGGGGCTGGCGGCTTG<br>CAGGCGGGCCGCAGCCGACGCCGAGACTGACAACCCGTG	H16_A0511

[a] Locus tag of downstream gene of each promoter

Supplementary Table S5. Target promoter-gene combinations for the qRT-PCR analysis

Promoter ID	Gene	Information [a]
PS01	*cbbL*	H16_B1395
PS01	*ccr_CA*	Integrated into chromosome 1 of *C. necator* strain DL_1A23
PS07	*hoxK*	PHG001
PS07	*mcl*	Integrated into chromosome 1 of *C. necator* strain DL_1A23
PS11	*hoxN*	PHG023
PS11	*lccPccB*	Integrated into chromosome 1 of *C. necator* strain DL_1A23

[a] Locus tag of endogenous genes or the information of the exogenous genes introduced into *C. necator*.

References

Alterio, V., Langella, E., De Simone, G., and Monti, S. M. (2015). Cadmium-containing carbonic anhydrase CDCA1 in marine diatom *Thalassiosira weissflogii*. *Mar. Drugs*. 13 (4), 1688–1697. doi: 10.3390/md13041688

Erb, T. J., Frerichs-Revermann, L., Fuchs, G., and Alber, B. E. (2010). The apparent malate synthase activity of *Rhodobacter sphaeroides* is due to two paralogous enzymes, (3S)-Malyl-coenzyme A (CoA)/{beta}-methylmalyl-CoA lyase and (3S)- Malyl-CoA thioesterase. *J. Bacteriol*. 192 (5), 1249–1258. doi: 10.1128/JB.01267-09

Erb, T. J., Rétey, J., Fuchs, G., and Alber, B. E. (2008). Ethylmalonyl-CoA mutase from *Rhodobacter sphaeroides* defines a new subclade of coenzyme B_{12}-dependent acyl-CoA mutases. *J. Biol. Chem*. 283 (47), 32283–32293. doi: 10.1074/jbc.M805527200

Hisano, T., Tsuge, T., Fukui, T., Iwata, T., Miki, K., and Doi, Y. (2003) Crystal structure of the (R)-specific enoyl-CoA hydratase from *Aeromonas caviae* involved in polyhydroxyalkanoate biosynthesis. *J. Biol. Chem*. 278 (1), 617–624. doi: 10.1074/jbc.M205484200

Insomphun, C., Xie, H., Mifune, J., Kawashima, Y., Orita, I., Nakamura, S., and Fukui, T. (2015). Improved artificial pathway for biosynthesis of poly(3-hydroxybutyrate-co-3-hydroxyhexanoate) with high C6-monomer composition from fructose in *Ralstonia eutropha*. *Metab. Eng*. 27, 38–45. doi: 10.1016/j.ymben.2014.10.006

Liu, X., Feng, X., Ding, Y., Gao, W., Xian, M., Wang, J., and Zhao, G. (2020). Characterization and directed evolution of propionyl-CoA carboxylase and its application in succinate biosynthetic pathway with two CO_2 fixation reactions. *Metab. Eng*. 62, 42–50. doi: 10.1016/j.ymben.2020.08.012

Schwander, T., Schada von Borzyskowski, L., Burgener, S., Cortina, N. S., and Erb, T. J. (2016). A synthetic pathway for the fixation of carbon dioxide in vitro. *Science*. 354 (6314), 900–904. doi: 10.1126/science.aah5237

Tran, T. H., Hsiao, Y. S., Jo, J., Chou, C. Y., Dietrich, L. E., Walz, T., and Tong, L. (2015). Structure and function of a single-chain, multi-domain long-chain acyl-CoA carboxylase. *Nature*. 518 (7537), 120–124. doi: 10.1038/nature13912
